# Molecular prevalence and subtyping of *Cryptosporidium* spp*.* in fecal samples collected from stray cats in İzmir, Turkey

**DOI:** 10.1186/s12917-022-03190-y

**Published:** 2022-03-07

**Authors:** Ahmet Efe Köseoğlu, Hüseyin Can, Muhammet Karakavuk, Mervenur Güvendi, Aysu Değirmenci Döşkaya, Pumla Bhekiwe Manyatsi, Mert Döşkaya, Adnan Yüksel Gürüz, Cemal Ün

**Affiliations:** 1 Faculty of Science Department of Biology Molecular Biology Section, Ege University, İzmir, Turkey; 2 Ege University Vaccine Development Application and Research Center, İzmir, Turkey; 3 Ege University Ödemiş Technical Training College, İzmir, Turkey; 4 Faculty of Medicine Department of Parasitology, Ege University, İzmir, Turkey

**Keywords:** *Cryptosporidium felis*, *18S rRNA*, *gp60*, XIXa, Cats, Turkey

## Abstract

**Background:**

*Cryptosporidium* spp. are obligate intracellular apicomplexan parasites transmitted to humans and other animals by contaminated water, food, or direct contact. They mainly cause gastrointestinal symptoms, although subclinical infections are also common. Cats are primarily infected by host-adapted *Cryptosporidium felis* while *C. parvum* and *C. muris* have also been detected in some cases. In this study, the molecular prevalence of *Cryptosporidium* spp. was investigated by screening 399 fecal samples collected from stray cats using nested PCR targeting the 18S rRNA gene for the first time in Turkey. Additionally, *Cryptosporidium* PCR-positive samples were genotyped by nested PCR- restriction fragment length polymorphism (RFLP), and subsequently, amplicons of 18S SSU rRNA were sequenced. They were further subtyped by amplification and sequencing of the *gp60* gene.

**Results:**

Among fecal samples screened, 12 of them (3%) were found to be *Cryptosporidium-positive*, and according to RFLP and sequencing of *18S rRNA* gene, all positive samples were identified as *C. felis*. Subtyping analyses at the *gp60* gene showed that *C. felis* isolates belonged to the XIXa subtype family, which are closely related to human subtypes of the parasite.

**Conclusions:**

The results of this study are important in terms of indicating the potential role of stray cats for transmission of *Cryptosporidium* spp. to humans or other animals. Also, the presence of XIXa, which is the dominant subtype family of *C. felis* in cats and humans was shown for the first time in stray cats of İzmir, Turkey.

## Background

*Cryptosporidium* spp. are obligate intracellular apicomplexan parasites distributed worldwide and infecting all vertebrates including humans and domestic animals [1–3]. The main transmission routes of *Cryptosporidium* are consumption of contaminated water and food or direct contact with infected animals and humans [3]. In humans, *Cryptosporidium* causes gastrointestinal disturbance presented with diarrhea, although subclinical cases are also common. It may cause serious disseminated infections in immunocompromised individuals [3]. *Cryptosporidium* infection is generally asymptomatic in cats but symptoms like diarrhea may occur especially in young and newborn kittens. Additionally, the degeneration of host epithelial cells, loss of microvilli, and atrophy of the villi often occur in severe infections [4].

Among the 19 *Cryptosporidium* species (*C. hominis*, *C. parvum C. meleagridis*, *C. canis*, *C. felis*, *C. ubiquitum*, *C. cuniculus*, *C. viatorum*, *C. muris*, *C. andersoni*, *C. erinacei*, *C. tyzzeri*, *C. bovis*, *C. suis*, *C. scrofarum*, *C. occultus*, *C. xiaoi*, *C. fayeri* and *C. ditrichi*) and 4 genotypes (*Cryptosporidium* chipmunk genotype I, mink genotype, skunk genotype and horse genotype), *C. hominis* and *C. parvum* are the most prevalently found in humans [3]. *C. parvum* is a zoonotic species whereas mainly anthroponotic *C. hominis* has been detected in different non-human animal species [5, 6]. *Cryptosporidium canis*, *C. felis*, *C. meleagridis*, and *C. ubiquitum* are other significant zoonotic pathogens causing diseases in humans [3]. In cats, host-adapted *C. felis* is the main *Cryptosporidium* species, although *C. parvum* and *C. muris* have also been detected [7–12].

During the diagnosis of *Cryptosporidium*, microscopic staining methods such as Ziehl–Neelsen, Kinyoun, and Giemsa are used to identify parasite oocysts in the feces [7]. Antigen detection methods targeting the surface proteins of the parasite in stool samples are also used [13]. Microscopic and antigen detection methods are not useful for species and genotype identification due to morphologically identical oocysts and shared antigens [8].

Molecular methods including polymerase chain reaction (PCR), DNA sequencing, and restriction fragment length polymorphism (RFLP) based on the analysis of the small subunit 18S rRNA gene are used to detect the parasite DNA, and identify species and genotypes in animal, human, and environmental samples [7]. During the species identification and genotyping of *Cryptosporidium* spp. by an RFLP method, an 830-bp fragment of the 18S rRNA gene is digested using SspI and VspI restriction enzymes [14–16]. Additionally, a gene encoding 60-kDa glycoprotein (*gp60*) in the subtyping of *C. parvum*, *C. hominis*, *C. meleagridis*, *C. ubiquitum*, *C. viatorum*, *C. fayeri*, *C. ryanae*, *C. canis* and *C. felis* has been used as a marker [3, 5, 17–20].

The study region İzmir harbors several stray cats that have close contact with humans and do not have sufficient veterinary care to prevent *Cryptosporidium* spp. transmission. Many important zoonotic parasites such as *Toxoplasma gondii*, *Leishmania* spp., and *Blastocystis* spp. have been detected in stray cats of İzmir in our previous studies [21–25]. This study initially investigated *Cryptosporidium* spp. in stray cats by a comprehensive PCR screening performed in 399 fecal samples. Secondly, species identification and subtyping analyses have been performed in *Cryptosporidium-positive* samples by RFLP and sequencing of the 18S rRNA and *gp60* genes.

## Results

### Molecular prevalence of *Cryptosporidium* spp.

**Fig. 1:**
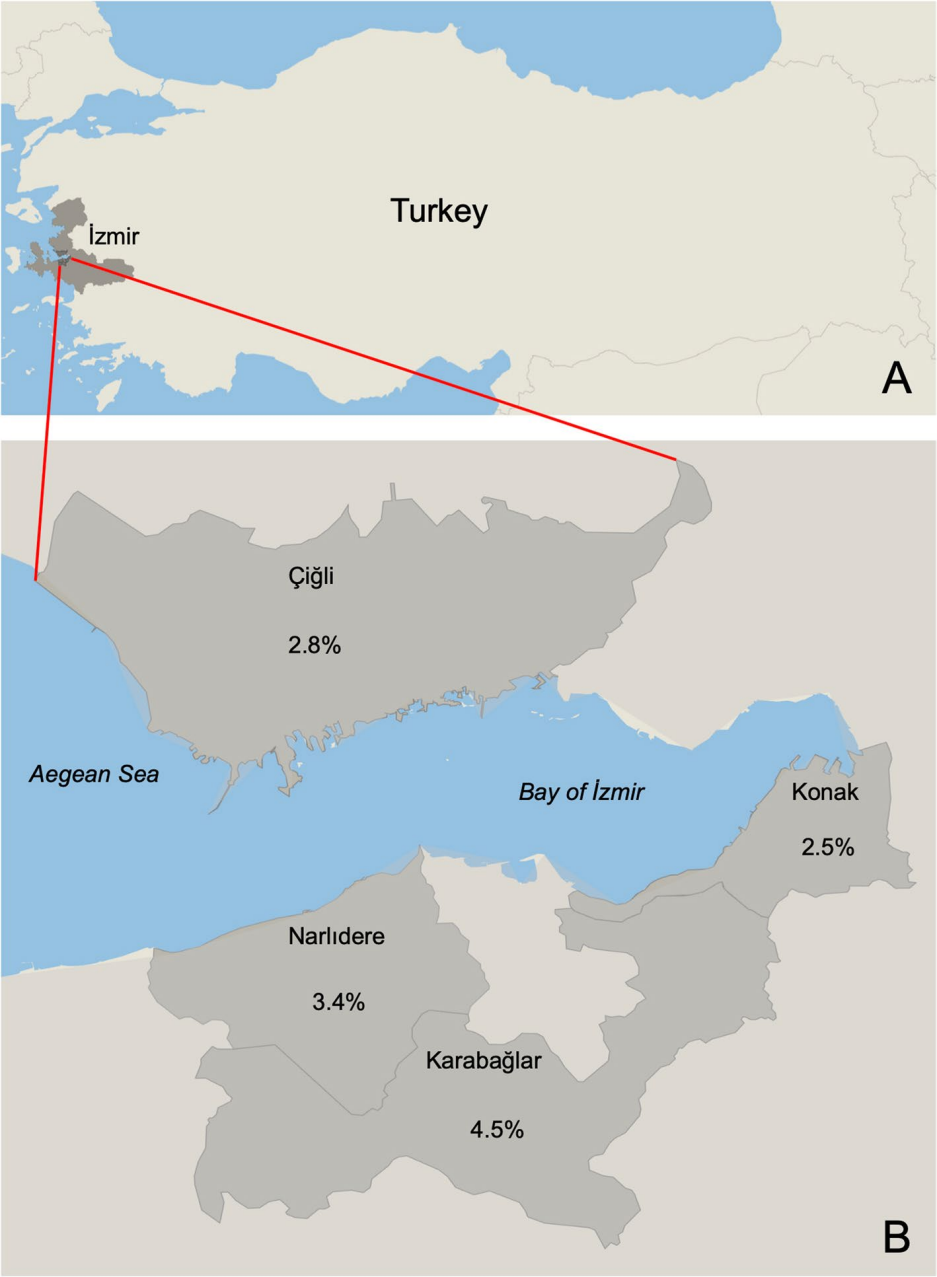
The map shows the prevalence values among different regions of Izmir, Turkey. Datawrapper (https://www.datawrapper.de/) was used to create the map

*Cryptosporidium* spp. DNA was detected in 12 of 399 fecal samples collected from stray cats by a nested PCR targeting the 18S rRNA gene. Accordingly, the molecular prevalence was 3%. Among the prevalence values of the regions, the highest prevalence value was observed in Karabağlar district with 4.5% which was followed by Narlıdere district with 3.4% (Fig. 1).

### Species identification

Species identification of *Cryptosporidium* PCR-positive samples was performed by two different approaches including RFLP and Sanger sequencing analyses. According to the results, all *Cryptosporidium-positive* samples were identified as *C. felis*. After digestion with SspI, two fragments 426 bp and 390 bp were detected for all *C. felis* positive samples. Three fragments 444 bp, 247 bp, and 106 bp were detected for *C. parvum* which was used as the positive control. Sequencing of 18S rRNA gene also confirmed that all *Cryptosporidium-positive* samples were *C. felis* (Table 1). Similarity rates of *C. felis* samples identified in this study varied between 91 and 98% compared to reference *C. felis* isolates deposited in GenBank (Table 1).

**Table 1:** BLAST results of *C. felis* isolates based on *18S rRNA* gene

Sample	Accession number	Percentage of nucleotide identity
*C. felis* cat isolate 1	KJ194110.1	725/780 (93%)
*C. felis* cat isolate 2	AB694730.1	468/491 (95%)
*C. felis* cat isolate 3	AB694730.1	658/722 (91%)
*C. felis* cat isolate 4	KJ194110.1	780/795 (98%)
*C. felis* cat isolate 5	KJ194110.1	774/794 (97%)
*C. felis* cat isolate 6	KJ194110.1	771/797 (97%)
*C. felis* cat isolate 7	KJ194110.1	768/806 (95%)
*C. felis* cat isolate 8	KJ194110.1	797/813 (98%)
*C. felis* cat isolate 9	MH115431.1	743/780 (95%)
*C. felis* cat isolate 10	MK982512.1	734/784 (94%)
*C. felis* cat isolate 11	AF159113.1	766/818 (94%)
*C. felis* cat isolate 12	AF159113.1	716/769 (93%)

### Subtyping of *C. felis*

**Fig. 2:**
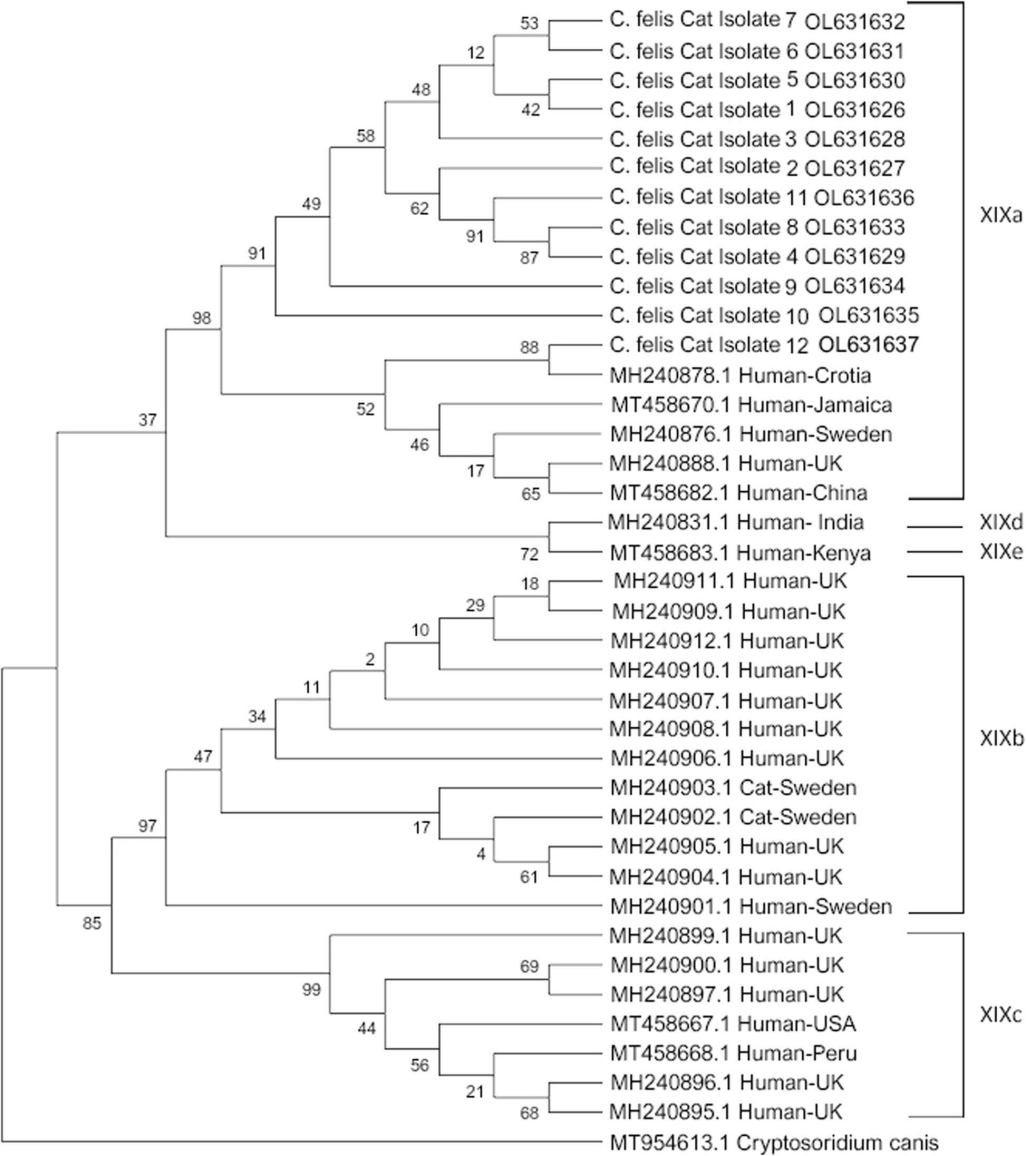
Phylogenetic tree shows *C. felis* isolates clustering with XIXa subtype family

Subtypes of *C. felis* isolates were identified by Sanger sequencing of the *gp60* gene. The scheme defined by Jiang et al., (2020) [26] was used for subtyping. The relation of subtypes was shown by phylogenetic analysis performed with reference *C. felis* isolates subtyped as XIXa, XIXb, XIXc, XIXd, or XIXe. According to this scheme, a repeat sequence with one copy called R1 (CCACCTAGTGGCGGTAGTGGCGTGTCCCCTGCT) and a 9 bp deletion were detected in all *C. felis* isolates. Also, two indels, which are located between positions 706-711 bp, and 1015–1068 bp, were not available. Another repeat sequence called R2 (AGCACAACTACGGCTACAGCGAGCACTGCGAGTTCGACA) was detected as one or two copies whereas the GGT sequence among positions 1156 and 1167 was detected as two, three, or four copies. These findings were compatible with the subtype family named as XIXa (Table 2). Also, all *C. felis* samples were clustered within the subtype family XIXa in the phylogenetic tree (Fig. 2).

**Table 2:** Subtyping of *C. felis* isolates using reference positions based on gp60 gene

Sample	R1 (463–556)	Deletion (667–679)	Indel (706–711)	R2 (770–910)	Indel (1015–1068)	Indel (1156–1167)	Subtype family
*C. felis* cat isolate 1	1 copy	9 bp	-	2 copy	-	4 GGT	XIXa
*C. felis* cat isolate 2	1 copy	9 bp	-	1 copy	-	^a^	XIXa
*C. felis* cat isolate 3	1 copy	9 bp	-	2 copy	-	^a^	XIXa
*C. felis* cat isolate 4	1 copy	9 bp	-	2 copy	-	^a^	XIXa
*C. felis* cat isolate 5	1 copy	9 bp	-	2 copy	-	3 GGT	XIXa
*C. felis* cat isolate 6	1 copy	9 bp	-	1 copy	-	4 GGT	XIXa
*C. felis* cat isolate 7	1 copy	9 bp	-	1 copy	-	4 GGT	XIXa
*C. felis* cat isolate 8	1 copy	9 bp	-	1 copy	-	2 GGT	XIXa
*C. felis* cat isolate 9	1 copy	9 bp	-	2 copy	-	3 GGT	XIXa
*C. felis* cat isolate 10	1 copy	9 bp	-	2 copy	-	2 GGT	XIXa
*C. felis* cat isolate 11	1 copy	9 bp	-	2 copy	-	4 GGT	XIXa
*C. felis* cat isolate 12	1 copy	9 bp	-	1 copy	-	4 GGT	XIXa
Reference isolate 1	1–3 copies	3 or 9 bp	-	1–4 copies	-	2–5 GGT	XIXa
Reference isolate 2	1 or 2 copies	6 or 15 bp	-	2–4 copies	18 bp	4 GGT	XIXb
Reference isolate 3	2 copies	15 bp	-	3 copies	-	3 GGT	XIXc
Reference isolate 4	1 copy	-	-	1 copy	-	4 GGT	XIXd
Reference isolate 5	1 copy	-	6 bp	1 copy	36 bp	4 GGT	XIXe

Reference positions are from Jiang et al. 2020 [26]

^a^shows the unevaluated sequences because of sequencing errors

R1: 33-bp tandem repeat (CCACCTAGTGGCGGTAGTGGCGTGTCCCCTGCT)

R2: 39-bp tandem repeat (AGCACAACTACGGCTACAGCGAGCACTGCGAGTTCGACA)

## Discussion

Cats are reservoirs of different infectious diseases caused by parasites or bacteria with zoonotic potential [27–29]. To date, the presence of important zoonotic parasites such as *T. gondii*, *Blastocystis* spp., and *Leishmania* spp. in stray cats living in İzmir (our study region) have been reported using molecular or serological methods with a prevalence of 14.4%, 3.7%, and 11.1%, respectively [22, 23, 25]. Although different *Cryptosporidium* species have been detected in cats, which play a role as reservoir host, the prevalence of *Cryptosporidium* spp., as well as species and subtypes in stray cats of İzmir are not known in detail. However, the prevalence of *Cryptosporidium* in humans has been frequently investigated by microscopy or immunological methods rather than molecular methods that enable species identification or subtyping in İzmir. According to obtained results, although the prevalence of *Cryptosporidium* spp. changed based on the sample number analyzed or method used, high prevalence rates varying from 33.5% to 37.4% were detected in İzmir [30, 31].

In this comprehensive study, three significant findings have been reported for the first time in Turkey. Firstly, the molecular prevalence of *Cryptosporidium* spp. was found to be 3% (12/399) in stray cats. Although this prevalence is low, it is rather important in terms of indicating that stray cats can act as a potential source of human and other animal infections in İzmir, Turkey. Secondly, all *Cryptosporidium-positive* samples were identified as *C. felis* by nested PCR–RFLP and Sanger sequencing. *Cryptosporidium felis* causes infections in cats and in some cases, in cows and humans as well [1, 3, 32]. Human cryptosporidiosis cases caused by *C. felis* have been detected frequently in developing countries and a zoonotic transmission from a household cat to its owner was reported [2, 17, 33–36]. However, there are uncertainties about the zoonotic transmission from cats into humans because of the limited number of cases and lack of sufficient information about *C. felis* isolated from cats at the genetic level [17]. In line with this, in a study investigating household transmission of zoonotic *Cryptosporidium* spp. from pet dogs and cats to their owners, it was emphasized that domestic cats do not play an important role as the source of human cryptosporidiosis [37].

The molecular prevalence of *C. felis* in cats varied from 0.3% to 11% in studies conducted in different regions (Table 3). Similarly, *C. felis* was detected in humans using molecular methods with different prevalence rates ranging from 0.4% to 4.5% (Table 3). Although any prevalence study associated with cryptosporidiosis in cats except our study showing a 3% *C. felis* prevalence has not been conducted in Turkey, a recent study identified for the first time a *C. felis* isolate (ANK_1) (NCBI accession number: MN394123)by molecular methods in a house cat [38]. Additionally, *C. felis* has been identified in surface water samples in the province of Samsun, Turkey [39]. This finding is relevant because it highlights the capability of infected cats of contaminating the environment (soil, waters) with their feces.

**Table 3:** Molecular prevalence and subtyping data of *C. felis* isolates detected in cats and humans from different countries

Molecular prevalence	Target gene	Subtyping gene	Subtype family	Host	Country	Reference
2.0% (7/357)	*18S rRNA*	-	-	household cats	Japan	[40]
0.3% (1/329)	*18S rRNA*	-	-	pet shop kittens	Japan	[40]
2.5% (2/80)	*18S rRNA*	-	-	cats	Thailand	[41]
4.5% (7/155)	*18S rRNA*	-	-	HIV-infected patients	Thailand	[42]
1.7% (18/1063)	*actin*	-	-	cats	Australia	[43]
11% (5/46)	*18S rRNA*	-	-	cats	Colombia	[44]
5.4% (3/55)	*18S rRNA*	-	-	cats	Brazil	[45]
4.8% (12/250)	*18S rRNA*	-	-	domestic cats	Mississippi and Alabama	[46]
1.92% (1/52)	*18S rRNA*	-	-	cats	China	[10]
5% (21/418)	*18S rRNA*	-	-	cats	China	[47]
0.49% (3/609)	*18S rRNA*	-	-	humans	China	[48]
0.35% (1/283)	*18S rRNA*	-	-	immunodeficient patients	Poland	[49]
3.3% (1/30)	*18S rRNA*	-	-	humans	Sweden	[50]
0.9% (1/108)	*18S rRNA*	-	-	patients	Mozambique	[51]
1% (4/398)	*18S rRNA*	*gp60*	XIXa	patients	Sweden	[52]
-	*18S rRNA*	*gp60*	XIXa	pet shelter/shop cats, stray cats	China	[17]
-	*18S rRNA*	*gp60*	XIXa	cats	Peru, USA, Slovakia, Australia	[26]
-	*18S rRNA*	*gp60*	XIXa, XIXc, XIXd, XIXe	humans	Peru, USA, Jamaica, Portugal, Nigeria, China, Kenya	[26]

In subtyping studies based on gp60 gene variations of *C. felis*, five subtype families (XIXa, XIXb, XIXc, XIXd, and XIXe) have been identified using 200 *C. felis* isolates from all around the world [17]. Among the five subtype families, the XIXa subtype family has been reported as the dominant subtype family in cats and humans in previous studies [17, 26, 52]. The third important finding in our study is the detection of subtype family XIXa in stray cats, which is consistent with previous results (Table 2; Fig. 2). This result also indicates that the XIXa subtype family is the dominant subtype in circulation causing the transmission between stray cats and humans in İzmir.

## Conclusion

The presence of *C. felis* in stray cats was demonstrated for the first time by analyzing a huge number of fecal samples in Turkey. Detection of the dominant XIXa subtype family in cats and humans indicated the possible transmission risk between stray cats and humans in İzmir. Accordingly, it was thought that the stool of stray cats should be analyzed for the presence of *C. felis* and positive stray cats should be treated to reduce the zoonotic transmission from cats to humans by the municipality veterinarians during their routine testing before sterilization operation.

## Materials and methods

### Fecal samples

Fecal samples (*n* = 399) collected from stray cats which were brought for routine surgical sterilization process performed by the Veterinary clinics of the Municipality of İzmir were included in this study. Regions that fecal samples were collected were Konak (*n* = 159), Narlıdere (*n* = 148), Çiğli (*n* = 70) and Karabağlar (*n* = 22) districts. Before surgical sterilization and receiving any treatment, the fecal samples were collected from stray cats which were kept in separate cages.

### Nested PCR–RFLP

DNA was extracted by a commercial stool DNA isolation kit (RTA Labs, Turkey) according to the manufacturer’s instructions. During DNA isolation, 100 mg of fecal sample was used, and they were eluted with 100 µl elution buffer [23]. A nested PCR targeting small subunit 18S rRNA gene was applied as described before [14]. In the initial reaction, 5'-TTCTAGAGCTAATACATGCG-3' and 5'-CCCTAATCCTTCGAAACAGGA-3' primers were used to amplify a 1325 bp gene fragment. In the second reaction, 5'-GGAAGGGTTGTATTTATTAGATAAAG-3' and 5'-AAGGAGTAAGGAACAACCTCCA-3' primers were used to amplify gene fragments with a molecular size approximately 826–864 bp from the initial reaction product. In the initial reaction of nested PCR, 100 μl amplification reaction included 2 μl template DNA, 2 μl primers, 16 μl MgCI_2_ (25 nM), 2 μl dNTP, 10 μl Taq buffer, and 0,5 μl Taq DNA Polymerase (ThermoFisher). In the second step of nested PCR, 4 μl PCR product as template DNA and 4 μl MgCI_2_ (25 nM) was used as different from the first step. The nested PCR was performed using the following protocol for both steps: 3 min initial denaturation step at 94 °C, followed by 35 cycles of 45 s at 94 °C, 45 s at 55 °C, and 1 min at 72 °C, and a final extension of 7 min at 72 °C. After amplification, PCR products were visualized using 1% agarose gel. Then, PCR products were purified by Qiaquick PCR Purification Kit (Qiagen, USA), sequenced, and compared with the NCBI database (https://www.ncbi.nlm.nih.gov) by BLAST (https://blast.ncbi.nlm.nih.gov/Blast.cgi). For sequencing of the 18S rRNA gene, forward primer (5'-GGAAGGGTTGTATTTATTAGATAAAG-3') was used.

For species identification of *Cryptosporidium-positive* samples, PCR products with a size of 826–864 bp were digested by SspI restriction enzyme (ThermoFisher) for 4 h [14–16]. During digestion, 20 μl total reaction volume included 10 μl amplified PCR product, 1.5 μl restriction enzyme, 2 μl restriction buffer (10x), and 6.5 μl distilled water. After digestion, PCR products were visualized using 2.5% agarose gel electrophoresis. After digesting by SspI enzyme, the expected product sizes for *C. felis* are 426 bp and 390 bp whereas [2, 16] the expected products for *C. parvum* are 444 bp, 247 bp, and 106 bp [16].

### Subtyping

*Cryptosporidium felis* isolates were subtyped by sequencing of the *gp60* gene as described previously [5]. In the initial reaction, GP60CF_F1 (5'-TTTCCGTTATTGTTGCAGTTGCA-3') and GP60CF_R1 (5'-ATCGGAATCCCACCATCGAAC-3') primers were used to amplify a 1200 bp gene fragment. In the second reaction, GP60CF_F2 (5'-GGGCGTTCTGAAGGATGTAA-3') and GP60CF_R2 (5'-CGGTGGTCTCCTCAGTCTTC-3') primers were used to amplify a 900 bp gene fragment from the initial reaction product. In the initial reaction of nested PCR, 20 μl amplification reaction included 3 μl template DNA, 0.5 μl primers, and 12.5 μl Dye Master Mix II (GeneMark). The second step of nested PCR was the same as the first step except the 1 μl PCR product that was used as template DNA. The nested PCR was performed using the following protocol for both steps: 4 min initial denaturation step at 95 °C, followed by 35 cycles of 30 s at 95 °C, 30 s at 55 °C, and 1.5 min at 72 °C, and a final extension of 7 min at 72 °C. Obtained PCR products were visualized using 1% agarose gel. Then, PCR products purified by Qiaquick PCR Purification Kit (Qiagen, USA) were sequenced, and generated sequences belonging to the gp60 gene were aligned with reference sequences subtyped as XIXa, XIXb, XIXc, XIXd, or XIXe by Jiang et al., 2020. For sequencing the *gp60* gene, forward primer (5'-GGGCGTTCTGAAGGATGTAA-3') was used. Subtype determination was performed by a scheme defined by Jiang et al. [26]. Next, a phylogenetic tree was constructed by the Maximum Likelihood method using the Kimura-3 parameter with 1000 bootstraps in order to confirm subtype results.

## Data Availability

All sequences obtained from *18S rRNA* and *gp60* genes of *Cryptosporidium* isolates were deposited into GenBank (National Center for Biotechnology Information Search database) under accession numbers OL615014- OL615025 (for the *18S rRNA* gene) and OL631626- OL631637 (for *gp60*).

## Abbreviations

*C. felis*: *Cryptosporidium felis*
*PCR*: Polymerase chain reaction
*gp60*: 60-KDa glycoprotein gene
*RFLP*: Restriction fragment length polymorphism
*18S** rRNA*: Small subunit rRNA gene
